# Supplementary material for: Cycle and flow trusses in directed networks
Source: R Soc Open Sci. 2016 Nov 30;3(11):160270. doi: 10.1098/rsos.160270 (PMC5180108; doi:10.1098/rsos.160270)
Supplement: A pdf file titled “supple_RSOS.pdf” which contains three supplementary figures and two supplementary tables [file rsos160270supp1.pdf]

Supplementary Materials

for

Taro Takaguchi and Yuichi Yoshida

Cycle and flow trusses in directed networks

S1 Algorithms to compute trusses

In this section, we describe our algorithm for finding the flow and cycle k-trusses in a network. For a network G, the sets of nodes and links are denoted by $V(G)$ and $E(G)$, respectively. For a node $v \in V(G)$, we define $N_G^+(v)$ as the set of out-neighbors of v, that is, $N_G^+(v) = \{w \in V(G) \mid vw \in E(G)\}$; we define $\deg_G^+(v)$ as the out-degree of v, i.e., $\deg_G^+(v) = |N_G^+(v)|$. Here, for the sake of simplicity, we denote the link from node u to node v by uv .

Our algorithm uses a subroutine called CommonNeighbor (Algorithm 1). This subroutine takes two networks G and G' on the same node set V and two nodes $u, v \in V$, and returns the set of nodes w such that $uw \in E(G)$ and $vw \in E(G')$. This subroutine will be used to enumerate the cycle or flow triangles involving the link uv. The algorithm is relatively straightforward: it chooses either u or v, enumerates its out-neighbors w, and then checks whether w is also an out-neighbor of the other unchosen node. For efficiency, we choose u if $\deg_G^+(u) < \deg_{G'}^+(v)$ and choose v otherwise. Using hash tables for storing out-neighbors of nodes, the time complexity of CommonNeighbor is bounded by $O(\min\{\deg_G^+(u), \deg_{G'}^+(v)\})$.

Algorithm 1

procedure CommonNeighbor(G, G', u, v) ▷ Find all w such that $uw \in E(G)$ and $vw \in E(G')$
  $W \leftarrow \emptyset$.
  if $\deg_G^+(u) < \deg_{G'}^+(v)$ **then**
    for $w \in N_G^+(u) \setminus \{v\}$ **do**
      if $vw \in E(G')$ **then**
        $W \leftarrow W \cup \{w\}$.
      end if
    end for
  else
    for $w \in N_{G'}^+(v) \setminus \{u\}$ **do**
      if $uw \in E(G)$ **then**
        $W \leftarrow W \cup \{w\}$.
      end if
    end for
  end if
  return W.
end procedure

Now we present our algorithm for enumerating cycle trusses (Algorithm 2). Given a network G, it computes cycle k-trusses for all k at once.

First, for every link $uv \in E(G)$, we count the number of cycle triangles involving uv and store the number to $c[uv]$ (Line 6). This can be done by calling CommonNeighbor(G', G, u, v), where G' is the network obtained from G by reversing the directions of links. This is because if there exists a cycle triangle in G with links uv, vw, and wu, then G' contains the link uw and G contains the link vw.

Algorithm 2

```
 1: procedure CycleTruss(G)                                   ▷ Find cycle-trusses in G
 2:     Let G′ be the network obtained from G by reversing the directions of links.
 3:     c[e] ← 0 for each e ∈ E(G).
 4:     ℓ[e] ← ∞ for each e ∈ E(G).
 5:     for uv ∈ E(G) do
 6:         c[uv] ← |CommonNeighbor(G′, G, u, v)|.      ▷ Count cycle triangles involving uv.
 7:     end for
 8:     k ← 0.
 9:     while a link remains do
10:         while there exists a link uv with c[uv] ≤ k do
11:             ℓ[uv] ← k.
12:             for w ∈ CommonNeighbor(G′, G, u, v) do
13:                 c[wu] ← c[wu] − 1 and c[vw] ← c[vw] − 1.
14:             end for
15:             Remove the link uv, and update G and G′.
16:         end while
17:         k ← k + 1.
18:     end while
19: end procedure
```

Next, starting with $k = 0$, as long as the links remain, we perform the following process: As long as there is a link uv such that $c[uv]$ is at most k, we set the truss number $\ell[uv]$ of uv to be k (Line 11), then for each cycle triangle involving the link uv, we decrease the count of the other two links (Line 12-14), and finally, we remove the link uv from the network (Line 15). If there is no link with a count of at most k, we increment the value of k and repeat the process. Note that, when the process starts for a particular value of k, all links have counts of at least k, and thus, all these links have truss numbers of at least k. On the other hand, when we remove a link in the process for a particular k value, because we have only removed links that cannot be a member of a cycle $(k+1)$-truss, its truss number is at most k. Therefore, each link is assigned with the correct truss number.

The overall time complexity of CycleTruss is dominated by the time complexity of enumerating the cycle triangles. Naively, this can be bounded by

$$\sum_{uv \in E(G)} \deg^{+}_{G}(v) = \sum_{v \in V(G)} \deg^{+}_{G}(v)^2 = O\Big(N^2 \cdot \frac{M}{N}\Big) = O(NM),$$

where N and M are the number of nodes and links, respectively, in the input network. In practice, however, the time taken runs is almost linear with M for real networks.

Finally, we explain our algorithm for enumerating flow trusses (Algorithm 3), which simultaneously computes flow k-trusses for all k values. Conceptually, FlowTruss is almost the same as CycleTruss. However, for a link uv and a node w, there are three types of a flow triangle involved: (i) a flow truss with links uv, wv, and uw, (ii) a flow truss with links uv, wv,

and wu, and (iii) a flow truss with links uv, vw, and wu. These links can be enumerated by calling CommonNeighbor(G, G'), CommonNeighbor(G', G'), and CommonNeighbor(G, G), respectively, where G' is the network obtained from G by reversing the directions of all links. The other parts of the algorithm and the analysis of time complexity are the same as those of CycleTruss, and therefore we omit them.

Algorithm 3

procedure FlowTruss(G) ▷ Find flow-trusses in G
    Let G' be the network obtained from G by reversing the directions of links.
    $c[e] \leftarrow 0$ for each $e \in E(G)$.
    $\ell[e] \leftarrow \infty$ for each $e \in E(G)$.
    for $uv \in E(G)$ **do**
        $c[uv] \leftarrow |$CommonNeighbor$(G, G', u, v)|$.
        ▷ Count flow triangles with links uv, wv, and uw for some $w \in V(G)$.
        $c[uv] \leftarrow c[uv] + |$CommonNeighbor$(G', G', u, v)|$.
        ▷ Count flow triangles with links uv, wv, and wu for some $w \in V(G)$.
        $c[uv] \leftarrow c[uv] + |$CommonNeighbor$(G, G, u, v)|$.
        ▷ Count flow triangles with links uv, vw, and uw for some $w \in V(G)$.
    end for
    $k \leftarrow 0$.
    while a link remains **do**
        while there exists a link uv with $c[uv] \le k$ **do**
            $\ell[uv] \leftarrow k$.
            for $w \in$ CommonNeighbor(G, G', u, v) **do**
                $c[uw] \leftarrow c[uw] - 1$ and $c[wv] \leftarrow c[wv] - 1$.
            end for
            for $w \in$ CommonNeighbor(G', G', u, v) **do**
                $c[wu] \leftarrow c[wu] - 1$ and $c[wv] \leftarrow c[wv] - 1$.
            end for
            for $w \in$ CommonNeighbor(G, G, u, v) **do**
                $c[wu] \leftarrow c[uw] - 1$ and $c[vw] \leftarrow c[vw] - 1$.
            end for
            Remove the link uv, and update G and G'.
        end while
        $k \leftarrow k + 1$.
    end while
end procedure

S2 Data sources

The network data sets used in the present study were downloaded from the following websites.

- The airport, communication, following, and software networks: `http://konect.uni-koblenz.de/`
- the USairport500 network: `http://toreopsahl.com/datasets/#usairports`
- The circuit networks and the word networks: `http://www.weizmann.ac.il/mcb/UriAlon/download/collection-complex-networks`
- The allcites network (the U.S. supreme court citation network): `http://fowler.ucsd.edu/judicial.htm`
- The cit-HepPh, cit-HepTh, social, slashdot-0902, twitter_combined, wiki-Vote, P2P, and the web networks: `http://snap.stanford.edu/data/`
- The food webs and the Edinburgh Associative Thesaurus: `http://vlado.fmf.uni-lj.si/pub/networks/data/`
- The gene regulatory networks; `http://info.gersteinlab.org/Hierarchy`
- The *Caenorhabditis elegans (C. elegans)* neural network: `http://www.wormatlas.org/neuronalwiring.html`
- The brain connectivity networks: `https://sites.google.com/site/bctnet/datasets`
- The mac95 network: `http://www.biological-networks.org/?page_id=25`
- The polblog network (the hyperlink network between weblogs on US politics): `http://www-personal.umich.edu/~mejn/netdata/`

The metabolic networks were based on those used in Ref. [1] and the network data were given by Kazuhiro Takemoto through personal communication. Any additional information from links, such as the weight, sign, or time stamp, were discarded from the network data. We also removed the self-loops and multiple links to make the networks simple.

The basic statistics of the truss structure for empirical networks obtained from 12 different fields are summarized in Tables. S1 and S2. Except for $k^{\mathrm{f}}_{\max}$ for the circuit networks and a few examples, almost all $k^{\mathrm{c}}_{\max}$ and $k^{\mathrm{f}}_{\max}$ are nontrivial. Additionally, the $k^{\mathrm{c}}_{\max}$ and $k^{\mathrm{f}}_{\max}$ values do not necessarily increase with the number of cycle and flow triangles. This result implies that the trusses can indicate the information regarding the module structure, irrespective of the count of these triangles.

Table S1: Statistics of the empirical network used in this study. N and M: the number of nodes and links. $k^{\mathrm{c}}_{\max}$ and $k^{\mathrm{f}}_{\max}$: the maximum cycle and flow truss numbers. C: the average clustering coefficient after discarding the link direction. T^{c} and T^{f}: the number of the cycle and flow triangles. p: the reciprocity of a network defined by the double of the total number of bidirectionally adjacent pairs divided by the total number of links.

name	N	M	$k^{\mathrm{c}}_{\max}$	$k^{\mathrm{f}}_{\max}$	C	T^{c}	T^{f}	p
**airport**								
openflights [2]	2939	30501	21	63	0.255	72631	72803	0.972
USairport500 [3]	500	5960	25	75	0.351	18424	18424	1
USairport_2010 [4]	1574	28236	54	163	0.384	220832	243384	0.781
**circuit**								
s208_st [5]	122	189	1	0	0.057	10	0	0
s420_st [5]	252	399	1	0	0.052	20	0	0
s838_st [5]	512	819	1	0	0.048	40	0	0
**citation**								
allcites [6, 7]	25417	216738	1	13	0.126	49	385667	0.003
cit-HepPh [8, 9]	34546	421534	1	23	0.146	506	1276803	0.003
cit-HepTh [8, 9]	27769	352768	3	28	0.120	522	1478675	0.003
**communication**								
email-EuAll [10]	265009	418956	12	39	0.004	134844	266308	0.260
enron [11]	86978	320154	13	50	0.072	255012	1171455	0.142
munmun_digg_reply [12]	30360	85247	1	2	0.006	286	4028	0.002
opsahl-ucsocial [13]	1899	20296	3	10	0.057	8441	14253	0.636
radoslaw_email [14]	168	11544	62	189	0.825	176867	203312	0.803
slashdot-threads [15]	51083	130370	2	5	0.006	4320	18175	0.212
wiki-Talk [16]	2394385	5021410	28	91	0.002	4302222	9031616	0.144
**following**								
munmun_twitter_social [17]	465017	834797	1	4	0.001	119	38375	0.003
polblogs [18]	1224	19022	9	32	0.226	18481	100562	0.243
soc-Epinions1 [19]	75879	508837	18	60	0.066	580213	1616825	0.405
soc-Slashdot0902 [20]	82168	870161	33	99	0.024	493487	602500	0.841
twitter_combined [21]	81306	1768135	41	139	0.171	5118668	13059341	0.482
wiki-Vote [22, 23]	7115	103689	6	25	0.126	41856	601594	0.056

Table S2: Statistics of the empirical network used in this study. See the caption of table S1 for the descriptions of the quantities.

name	N	M	$k^{\mathrm{c}}_{\max}$	$k^{\mathrm{f}}_{\max}$	C	T^{c}	T^{f}	p
**food web**								
Chesapeake [24]	39	176	1	3	0.284	14	194	0.068
ChesLower [25]	37	177	1	4	0.353	24	241	0.113
ChesMiddle [25]	37	207	1	6	0.432	38	383	0.087
ChesUpper [25]	37	214	1	5	0.420	44	393	0.140
CrystalC [26, 27]	24	125	1	5	0.493	41	209	0.176
CrystalD [26, 27]	24	99	1	4	0.394	24	127	0.141
Everglades [28]	69	911	2	10	0.470	536	4344	0.068
Florida [29]	128	2106	1	9	0.312	357	8367	0.029
Maspalomas [30]	24	82	1	2	0.318	9	59	0.122
Michigan [31]	39	218	1	4	0.335	52	332	0.083
Mondego [32]	46	392	1	9	0.491	224	1185	0.173
Narragan [33]	35	218	1	5	0.443	69	446	0.128
Rhode [34]	19	53	0	2	0.255	0	22	0.302
StMarks [35]	54	353	1	5	0.333	15	650	0.017
**gene**								
Hs_Tr [36]	3107	6873	1	5	0.008	46	2690	0.010
Mm_Tr [36]	1192	2393	1	2	0.009	1	206	0
Mt_Tr [36]	755	887	0	2	0.005	0	49	0.007
Rr_Tr [36]	533	1089	0	1	0.012	0	102	0.006
Sc_Tr [36]	4441	12873	1	4	0.010	13	3742	0.001
**P2P**								
p2p-Gnutella04 [10, 37]	10876	39994	1	2	0.005	33	901	0
p2p-Gnutella31 [10, 37]	62586	147892	1	2	0.004	57	1967	0
**neural**								
cat [38]	95	2126	8	25	0.489	5367	5929	0.899
celegans_neural [39]	279	2990	3	9	0.214	1414	4408	0.470
fve32 [40]	32	315	4	13	0.581	380	486	0.768
macaque71 [41]	71	746	4	12	0.442	813	957	0.826
mac95 [42, 43]	94	2390	15	47	0.793	9145	13802	0.732
**software**								
subelj_jdk [44]	6434	53892	2	16	0.011	288	194798	0.009
subelj_jung-j [44]	6210	50535	4	16	0.011	300	182009	0.010
**web**								
web-BerkStan [20]	685224	7600545	161	483	0.007	7426999	64666756	0.250
web-Google [20]	875713	5105039	31	93	0.055	2486567	13357485	0.307
web-NotreDame [45]	325729	1469679	148	444	0.088	6936636	8900531	0.517
web-Stanford [20]	281903	2312497	40	120	0.009	689426	11320457	0.277
**word**								
darwinbookinter_st [46]	7381	46281	7	22	0.036	63392	144954	0.090
EAT [47]	23132	311758	2	8	0.040	49884	395238	0.094
frenchbookinter_st [46]	8325	24295	3	7	0.012	6543	15834	0.037
japanesebookinter_st [46]	2704	8300	3	9	0.030	3194	7320	0.073
lasagne-spanishbook [44]	12643	57451	5	21	0.009	47110	110228	0.085
lasagne-yahoo [44]	653260	2931706	1	1	5.2×10^{-6}	4	67256	5.5×10^{-6}
spanishbookinter_st [46]	11586	45129	5	21	0.017	40868	97112	0.091

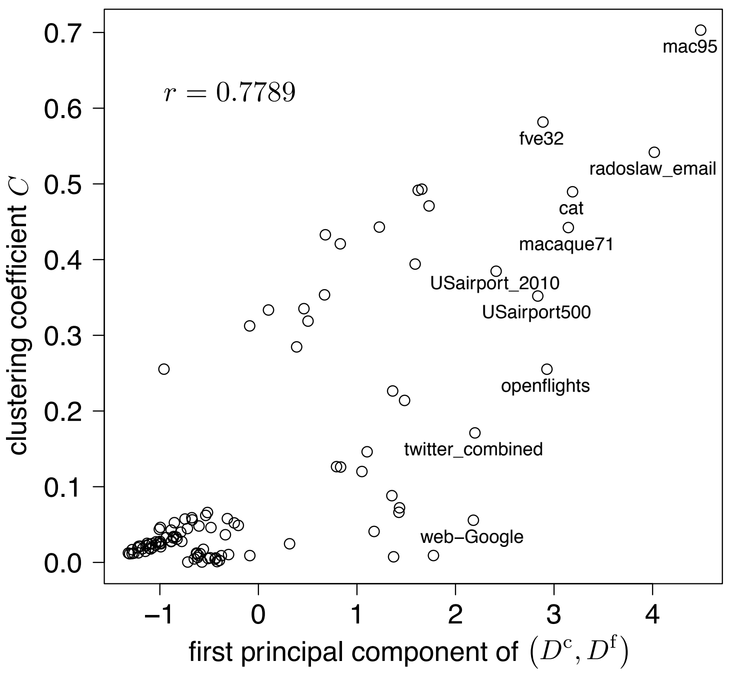

Figure S1: **Scatter plot of the values of the first principal component of $\left(D^{c}, D^{f}\right)$ and the clustering coefficient for various networks.** The clustering coefficient is calculated with regarding networks as undirected. The points corresponding to the ten networks with the largest values of the principal component are indicated with the names of networks. The r value is the Pearson correlation coefficient.

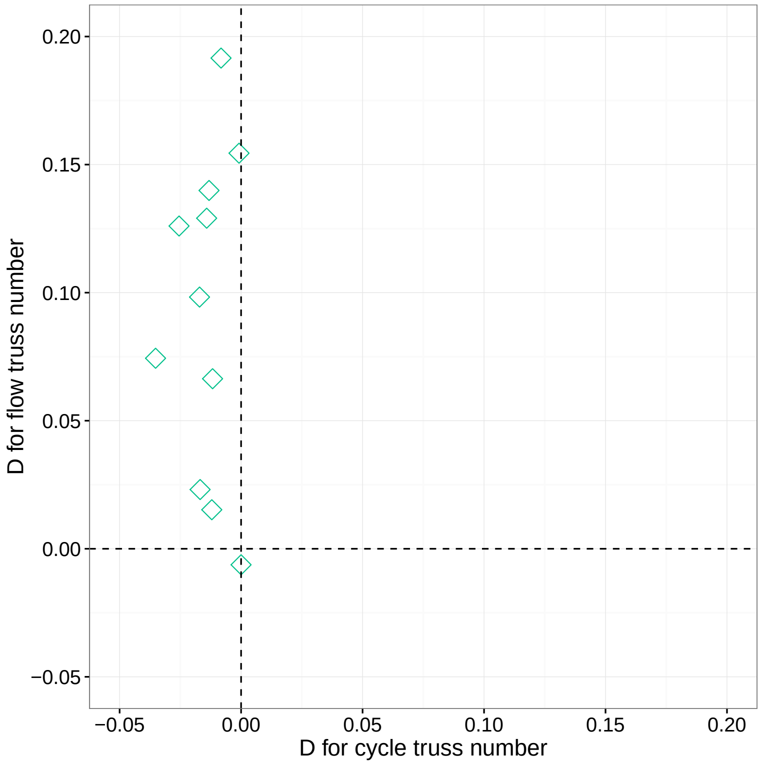

Figure S2: **Scatter plot of D^{c} and D^{f} for the food web networks.** The randomization method of networks [48] that retains the number of the cycle triangles and that of the flow triangles is applied.

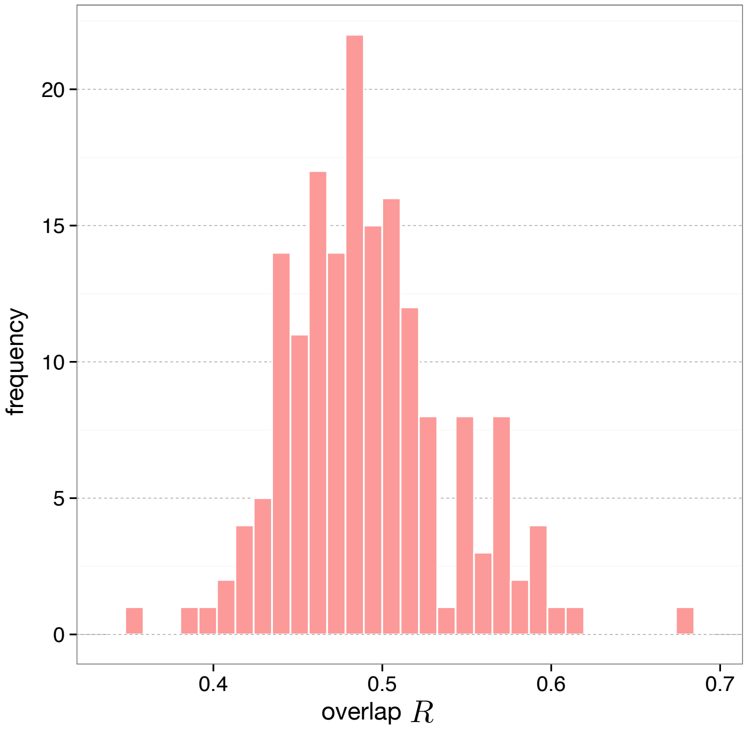

Figure S3: **Histogram of the R measure for the metabolic networks.** The measure R quantifies the overlap between the set of links with the largest k^{c} values and that with the largest k^{f} values.

References

[1] Takemoto K. 2014 Metabolic networks are almost nonfractal: A comprehensive evaluation. *Phys. Rev. E* **90**, 022802.

[2] Opsahl T, Agneessens F, Skvoretz J. 2010 Node centrality in weighted networks: Generalizing degree and shortest paths. *Soc. Networks* **32**, 245–251.

[3] Colizza V, Pastor-Satorras R, Vespignani A. 2007 Reaction-diffusion processes and metapopulation models in heterogeneous networks. *Nat. Phys.* **3**, 276–282.

[4] Opsahl T. 2011 Why Anchorage is not (that) important: Binary ties and Sample selection, `http://toreopsahl.com/2011/08/12/why-anchorage-is-not-that-important-binary-ties-and-sample-selection/` (Date accessed: 1st March 2016).

[5] Milo R, Shen-Orr S, Itzkovitz S, Kashtan N, Chklovskii D, Alon U. 2002 Network motifs: simple building blocks of complex networks. *Science (New York, N.Y.)* **298**, 824–827.

[6] Fowler JH, Johnson TR, Spriggs JF, Jeon S, Wahlbeck PJ. 2007 Network analysis and the law: Measuring the legal importance of precedents at the U.S. supreme court. *Polit. Anal.* **15**, 324–346.

[7] Fowler JH, Jeon S. 2008 The authority of Supreme Court precedent. *Soc. Networks* **30**, 16–30.

[8] Gehrke J, Ginsparg P, Kleinberg J. 2003 Overview of the 2003 KDD Cup. *ACM SIGKDD Explor.* **5**, 149.

[9] Leskovec J, Kleinberg J, Faloutsos C. 2005 Graphs over time: Densification laws, shrinking diameters and possible explanations. In *Proc. the eleventh ACM SIGKDD international conference on Knowledge discovery in data mining*, Chicago, IL, USA, 21 to 24 August 2005, pp. 177–187.

[10] Leskovec J, Kleinberg J, Faloutsos C. 2007 Graph evolution: Densification and shrinking diameters. *ACM Trans. Knowl. Discov. Data* **1**, 2.

[11] Klimt B, Yang Y. 2004 The Enron corpus: A new dataset for email classification research. In *Proc. 15th European Conference on Machine Learning*, Pisa, Italy, 20 to 24 September 2004, pp. 217–226.

[12] De Choudhury M, Sundaram H, John A, Seligmann DD. 2009 Social synchrony: Predicting mimicry of user actions in online social media. In *Proc. International Conference on Computational Science and Engineering* **4**, Vancouver, BC, Canada, 29 to 31 August 2009, pp. 151–158.

[13] Opsahl T, Panzarasa P. 2009 Clustering in weighted networks. *Soc. Networks* **31**, 155–163.

[14] Michalski R, Palus S, Kazienko P. 2011 Matching organizational structure and social network extracted from email communication. In *Proc. 14th International Conference on Business Information Systems*, Poźan, Poland, 15 to 17 June 2011, pp. 197–206.

[15] Gómez V, Kaltenbrunner A, López V. 2008 Statistical analysis of the social network and discussion threads in slashdot. In *Proc. the 17th international conference on World Wide Web*, Beijing, China, 21 to 25 April 2008, pp. 645–654.

[16] Leskovec J, Huttenlocher D, Kleinberg J. 2008 Governance in social media: A case study of the Wikipedia promotion process. In *Proc. Fourth International AAAI Conference on Weblogs and Social Media*, Washington, D.C., USA, 23 to 26 May 2010, pp. 98–105.

[17] De Choudhury M, Lin Y-R, Sundaram H, Candan KS, Xie L, Kelliher A. 2010 How does the data sampling strategy impact the discovery of information diffusion in social media? In *Proc. Fourth International AAAI Conference on Weblogs and Social Media*, Washington, D.C., USA, 23 to 26 May 2010, pp. 34–41.

[18] Adamic LA, Glance N. 2005 The political blogosphere and the 2004 US election: divided they blog. In *Proc. the 3rd international workshop on Link discovery*, Chicago, IL, USA, 21 to 24 August 2005, pp. 36–43.

[19] Richardson M, Agrawal R, Domingos P. 2003 Trust management for the semantic web. In *Proc. the Second International Semantic Web Conference*, Sanibel Island, FL, USA, 20 to 23 October 2003, pp. 351–368.

[20] Leskovec J, Lang KJ, Dasgupta A, Mahoney MW. 2009 Community structure in large networks: Natural cluster sizes and the absence of large well-defined clusters. *Internet Math.* **6**, 29–123 (2009).

[21] McAuley J, Leskovec J. 2014 Discovering social circles in ego networks. *ACM Trans. Knowl. Discov. Data* **8**, 1–28.

[22] Leskovec J, Huttenlocher D, Kleinberg J. 2010 Signed networks in social media. In *Proc. the SIGCHI Conference on Human Factors in Computing Systems*, Atlanta, GA, USA, 10 to 15 April 2010, pp. 1361–1370.

[23] Leskovec J, Huttenlocher D, Kleinberg J. 2010 Predicting positive and negative links in online social networks. In *Proc. the 19th international conference on World wide web*, Raleigh, NC, USA, 26 to 30 April 2010, pp. 641–650.

[24] Baird D, Ulanowicz RE. 1989 The seasonal dynamics of the Chesapeake Bay ecosystem. *Ecol. Monogr.* **59**, 329.

[25] Hagy JD. 2002 *Eutrophication, hypoxia and trophic transfer efficiency in Chesapeake Bay.* PhD thesis, University of Maryland.

[26] Homer M, Kemp WM. Unpublished manuscript.

[27] Ulanowicz RE. 1986 *Growth and Development: Ecosystems Phenomenology.* New York, NY: Springer New York.

[28] Ulanowicz RE, Heymans JJ, Egnotoivich MS. 2000 Network analysis of trophic dynamics in south Florida ecosystems, FY 99: The Graminoid ecosystem. Technical report, Center for Environmental Science, the University of Maryland, Solomons, MD.

[29] Ulanowicz RE, Bondavalli C, Egnotovich MS. 1998 Network analysis of trophic dynamics in south Florida ecosystem, FY 97: The Florida Bay ecosystem. Technical report, Chesapeake Biological Laboratory, University of Maryland, Solomons, MD.

[30] Almunia J, Basterretxea G, Arístegui J, Ulanowicz RE. 1999 Benthic-pelagic switching in a coastal subtropical lagoon. *Estuar. Coast. Shelf Sci.* **49**, 363–384.

[31] Krause AE. 2004 *The role of compartments in food-web structure and changes following biological invasions in southeast Lake Michigan.* Ph.D thesis, Michigan State University.

[32] Patricio J. 2002 Master's thesis, University of Coimbra, Portugal.

[33] Monaco ME, Ulanowicz RE. 1997 Comparative ecosystem trophic structure of three U.S. mid-Atlantic estuaries. *Mar. Ecol. Prog. Ser.* **161**, 239–254.

[34] Correll D. Unpublished manuscript.

[35] Baird D, Luczkovich J, Christian RR. 1998 Assessment of spatial and temporal variability in ecosystem attributes of the St Marks national wildlife refuge, Apalachee Bay, Florida. *Estuar. Coast. Shelf Sci.* **47**, 329–349.

[36] Bhardwaj N, Yan K-K, Gerstein MB. 2010 Analysis of diverse regulatory networks in a hierarchical context shows consistent tendencies for collaboration in the middle levels. *Proc. Natl. Acad. Sci. U.S.A.* **107**, 6841–6846.

[37] Ripeanu M, Foster I. 2002 Mapping the Gnutella network: Macroscopic properties of large-scale peer-to-peer systems. *IEEE Internet Comput. J.*, 6.

[38] Scannell JW, Burns GAPC, Hilgetag CC, O'Neil MA, Young MP. 1999 The connectional organization of the cortico-thalamic system of the cat. *Cereb. Cortex* **9**, 277–299.

[39] Varshney LR, Chen BL, Paniagua E, Hall DH, Chklovskii DB. 2011 Structural properties of the Caenorhabditis elegans neuronal network. *PLOS Comput. Biol.* **7**, e1001066.

[40] Felleman DJ, Van Essen DC. 1991 Distributed hierarchical processing in the primate cerebral cortex. *Cereb. Cortex* **1**, 1–47.

[41] Young MP. 1993 The organization of neural systems in the primate cerebral cortex. *Proc. R. Soc. B* **252**, 13–18.

[42] Kaiser M, Hilgetag CC. 2006 Nonoptimal component placement, but short processing paths, due to long-distance projections in neural systems. *PLOS Comput. Biol.* **2**, e95.

[43] Kötter R. 2004 Online retrieval, processing, and visualization of primate connectivity data from the CoCoMac database. *Neuroinformatics* **2**, 27–144.

[44] KONECT: the Koblenz Network Collection. `http://konect.uni-koblenz.de/` (Date accessed: 1st March 2016).

[45] Albert R, Barabási A-L, Jeong H. 1999 Diameter of the World-Wide Web. *Nature* **401**,130–131.

[46] Milo R, Itzkovitz S, Kashtan N, Levitt R, Shen-Orr S, Ayzenshtat I, Sheffer M, Alon U. 2004 Superfamilies of evolved and designed networks. *Science (New York, N.Y.)* **303**, 1538–1542.

[47] Kiss GR, Armstrong C, Milroy R, Piper J. 1973 An associative thesaurus of English and its computer analysis. In *The computer and literary studies* (eds. Aitken AJ, Bailey RW, Hamilton-Smith N), pp. 153–165. Edinburgh, UK: Edinburgh University Press.

[48] Michoel T, Joshi A, Nachtergaele B, Van de Peer Y. 2011 Enrichment and aggregation of topological motifs are independent organizational principles of integrated interaction networks. *Mol. Biosyst.* **7**, 2769–2778.
